# Nrf2/NOX2 Pathway Dysregulation and Oxidative Stress Biomarkers in Gaucher Disease–Associated Parkinsonism: Insights Into a Potential Therapeutic Target

**DOI:** 10.1111/jcmm.71225

**Published:** 2026-06-02

**Authors:** Alessio Ardizzone, Marika Lanza, Anna Paola Capra, Giovanna Casili, Maria Bulzomì, Fabiola De Luca, Irene Paterniti, Michela Campolo, Emanuela Esposito

**Affiliations:** ^1^ UniCamillus‐Saint Camillus International University of Health Sciences Rome Italy; ^2^ Department of Chemical, Biological, Pharmaceutical and Environmental Sciences University of Messina Messina Italy

**Keywords:** Gaucher disease (GD), Nrf2/NOX2 signalling axis, oxidative stress, parkinsonism

## Abstract

Parkinson's disease (PD) is the second most prevalent neurodegenerative disorder, yet its underlying genetic and molecular mechanisms remain incompletely understood. Variants in the *GBA* gene, encoding the lysosomal enzyme glucocerebrosidase, are not only responsible for Gaucher disease (GD) but also represent a significant genetic risk factor for PD, contributing to lysosomal dysfunction, oxidative stress and autophagy impairment. Among the key regulators of redox homeostasis, the Nrf2/NOX2 signalling axis has emerged as a pivotal pathway in the modulation of neuroinflammation and neurodegeneration. This study aims to explore the pathogenic link between *GBA* mutations and PD, focusing on the redox imbalance and the role of Nrf2 signalling in an in vivo *Gba* D409V knock‐in (KI) mouse model, compared to wild‐type (WT) C57BL/6J controls. Animals 8‐weeks old were evaluated over a 3‐month period, with tissue and behavioural assessments conducted at 7, 14, 30, 60 and 90 days. Early timepoints (7 and 14 days) did not reveal significant changes in behavioural performance, expression of PD‐related markers (TH, DAT, α‐synuclein), or oxidative stress indicators, including Nrf2, NOX2, malondialdehyde (MDA) and nitrate/nitrite levels. However, at 30, 60 and especially 90 days, significant alterations emerged, particularly a disrupted Nrf2/NOX2 balance, accompanied by molecular and biochemical signatures of oxidative stress. These findings suggest a time‐dependent progression of oxidative alterations in this GD model and support the role of *GBA* variants in promoting neurodegenerative processes. Unravelling these mechanisms is essential for the identification of early biomarkers and may offer new therapeutic insights for *GBA1*‐associated PD.

## 1 Introduction

Gaucher disease (GD) is the most common lysosomal storage disorder and is caused by mutations in the *GBA1* gene, which encodes glucocerebrosidase (GCase), the lysosomal enzyme responsible for hydrolyzing glucosylceramide into glucose and ceramide [1]. Loss‐of‐function mutations result in defective enzymatic activity, leading to the accumulation of glucosylceramide and glucosylsphingosine within lysosomes [1]. The pathophysiological consequences of this storage are multifaceted, including lysosomal dysfunction, impaired autophagy, inflammation and secondary mitochondrial abnormalities [1].

From an epidemiological perspective, GD has a global incidence estimated at approximately 1 in 40,000 to 1 in 60,000 live births, although this figure varies considerably depending on ethnic background [2]. The highest prevalence is observed among individuals of Ashkenazi Jewish descent, with an incidence that rises to about 1 in 850 [3].

GD affects males and females equally, with no gender predilection, as expected for an autosomal recessive disorder [4]. GD disease spectrum is heterogeneous, ranging from the non‐neuronopathic type I, which accounts for more than 90% of cases in Western countries, to the rarer neuronopathic types II and III, which manifest with severe neurological involvement [5]. Furthermore, advances in newborn screening programmes and genetic testing have also revealed a higher‐than‐expected frequency of asymptomatic or mildly symptomatic carriers, expanding the recognized epidemiological footprint of GD [6].

Therapeutically, major advances have transformed the clinical management of GD type I. Enzyme replacement therapy (ERT), using recombinant GCase analogues such as imiglucerase, velaglucerase alfa and taliglucerase alfa, has become the standard of care, effectively reducing hepatosplenomegaly, improving haematological parameters, and enhancing quality of life [7]. Substrate reduction therapy (SRT), with oral agents such as miglustat and eliglustat, offers an alternative approach by decreasing the synthesis of glucosylceramide [8]. While these therapies have markedly improved outcomes in non‐neuronopathic GD, they remain largely ineffective in types II and III due to the inability of current formulations to cross the blood–brain barrier [9, 10]. As such, neuroprotective strategies and innovative approaches, including small‐molecule chaperones and gene therapy, are under investigation to address the neurological manifestations that remain an unmet clinical need [10].

Over the last two decades, a growing body of clinical, genetic and neuropathological evidence has firmly established a link between Gaucher disease (GD) and Parkinson's disease (PD) [11].

Epidemiological studies initially noted a higher prevalence of parkinsonian features in GD patients and their relatives, prompting systematic genetic investigations [12]. These studies revealed that heterozygous *GBA* mutations represent the most common genetic risk factor for PD, conferring a 5‐ to 10‐fold increased risk of disease development and often being associated with earlier onset, faster progression and more severe non‐motor symptoms compared to sporadic PD [13].

Neuropathological investigations further supported this association, showing that both GD patients and asymptomatic *GBA* mutation carriers display an increased burden of α‐synuclein aggregates in the substantia nigra and other vulnerable brain regions [14]. While impaired lysosomal degradation of α‐synuclein resulting from GCase deficiency is thought to facilitate its misfolding and aggregation, accumulating evidence indicates that this relationship is bidirectional. In particular, aggregated α‐synuclein has been shown to further impair the trafficking and enzymatic activity of residual GCase, thereby exacerbating lysosomal dysfunction and reinforcing a pathogenic feed‐forward loop [15]. This reciprocal interaction is now recognized as a central mechanism linking *GBA* mutations to progressive synucleinopathy.

Complementary insights have come from experimental models. In cellular and murine systems, *GBA* mutations exacerbate dopaminergic neuron vulnerability, impair proteostasis and enhance mitochondrial dysfunction [13]. These alterations converge to compromise neuronal resilience under stress conditions. Despite these advances, the precise mechanisms by which defective GCase activity and α‐synuclein pathology cooperate to drive neurodegeneration remain incompletely understood. Among the multiple hypotheses, oxidative stress has emerged as a central pathogenic event, acting as a common denominator linking lysosomal dysfunction, mitochondrial abnormalities and α‐synuclein accumulation [16].

Oxidative stress represents a major hallmark of both GD and PD. The accumulation of storage lipids and α‐synuclein aggregation contributes to mitochondrial dysfunction and excessive production of reactive oxygen species (ROS) [17]. In parallel, neuroinflammatory responses may amplify the generation of reactive nitrogen species (RNS), further destabilizing cellular redox homeostasis [18]. Studies in patient‐derived samples and animal models have demonstrated elevated indices of lipid peroxidation, protein nitration and DNA oxidation, pointing to a state of persistent oxidative and nitrosative stress [19].

This redox imbalance not only compromises cellular macromolecules but also activates maladaptive signalling pathways, ultimately promoting neuronal dysfunction and death.

Two molecular players, nuclear factor erythroid 2–related factor 2 (Nrf2) and NADPH oxidase 2 (NOX2), could be particularly relevant in this context. Nrf2 is a transcription factor that orchestrates the expression of a wide range of antioxidant and cytoprotective genes, thereby serving as a master regulator of redox balance [20]. In contrast, NOX2 is a membrane‐bound enzyme complex that generates superoxide as part of immune and stress responses [21]. Under physiological conditions, a balance between Nrf2‐mediated defences and NOX2‐derived ROS production ensures redox homeostasis [22]. However, in pathological settings, this equilibrium can be disrupted. Reduced Nrf2 activity or overactivation of NOX2 has been associated with enhanced oxidative stress, neuroinflammation and neuronal loss [23].

An imbalance between Nrf2 and NOX2 has been described in various central nervous system (CNS) disorders [24]. In our previous work on migraine, we demonstrated that alterations in this axis contribute to the persistence of oxidative stress and neuronal hyperexcitability [25]. Similar mechanisms may also operate in GD and GD‐associated parkinsonism, where lysosomal and mitochondrial dysfunction converge to amplify ROS production and weaken antioxidant defences. Exploring the interplay between Nrf2 and NOX2 in these conditions could thus provide novel insights into shared pathogenic pathways and potential therapeutic strategies.

Based on these considerations, the present study was designed to investigate oxidative and nitrosative stress in the substantia nigra of *Gba* D409V knock‐in (KI) mice, a genetic line that recapitulates key features associated with GD‐related parkinsonism and is therefore commonly used for mechanistic studies. Specifically, we monitored the progression of Parkinson‐like features over time in *Gba* D409V KI mice, with the goal of capturing the temporal dynamics of redox alterations emerging during disease development. Particular attention was given to the Nrf2–NOX2 axis, whose dysregulation may act as a critical driver of oxidative stress. By characterizing when and how these pathways become perturbed, our aim was to identify Nrf2 as a potential predictive marker and therapeutic target in the context of *GBA*‐associated neurodegeneration.

## 2 Materials and Methods

### 2.1 Materials

Except where stated, reagents were supplied by Sigma‐Aldrich Company Ltd. (Milan, Italy) at the highest available commercial purity. Stock solutions were prepared using sterile, non‐pyrogenic saline (0.9% NaCl; Baxter, Italy/United Kingdom).

### 2.2 Animals

Male *Gba* D409V KI (C57BL/6N‐Gba1^tm1.1Mjff^/J; RRID:IMSR_JAX:019106) and C57BL/6J mice as wild‐type (WT; RRID:IMSR_JAX:000664) (8 weeks old, 25–30 g; The Jackson Laboratory) were maintained under controlled laboratory conditions (22°C ± 2°C, 55% ± 15% relative humidity, 12 h light/dark cycle), with ad libitum access to standard chow and water. Prior to the experiment, animals were housed in a quarantine area for 1 week and monitored to ensure suitability for the study. At each experimental time point, animals were randomly allocated to the experimental groups using a simple randomization procedure as described in next paragraph.

All procedures were carried out in accordance with Italian legislation on the protection of animals used for scientific purposes and Directive 2010/63/EU. The experimental protocol was approved by the Italian Ministry of Health (authorization n° 486/2024‐PR).

### 2.3 Experimental Design and Groups

**FIGURE 1:**
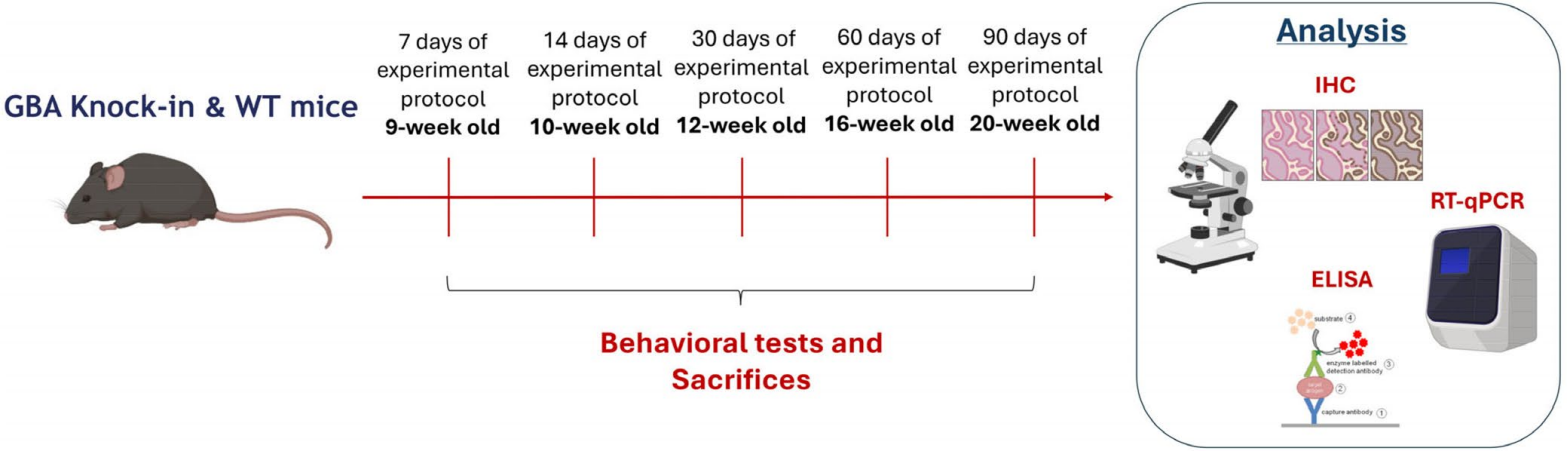
Schematic representation of the study design used to investigate the temporal progression of molecular and behavioural alterations in *Gba* D409V KI mice compared to WT (C57BL/6J) controls.

The study was designed to investigate the temporal progression of molecular and behavioural alterations in *Gba* D409V KI mice compared to WT (C57BL/6J) controls. Eight‐week‐old animals were monitored over a 3‐month period and sacrificed at predefined time points: 7, 14, 30, 60 and 90 days from the start of the experiment as detailed in Figure 1.

Animals (WT and KI) were randomly allocated to the experimental groups using a simple randomization procedure, ensuring balanced representation of genotypes across conditions.

*Gba D409V KI 9‐week‐old*: mice monitored at an early stage to detect potential initial molecular or behavioural changes at 9‐week‐old age (*n* = 9).

*C57BL/6J WT 9‐week‐old*: baseline reference group at 9‐week‐old age (*n* = 9).

*Gba D409V KI 10‐week‐old*: mice monitored at an early stage to detect potential initial molecular or behavioural changes at 10‐week‐old age (*n* = 9).

*C57BL/6J WT 10‐week‐old*: used for comparison with mutant mice at 10‐week‐old age (*n* = 9).

*Gba D409V KI 12‐week‐old*: mice monitored at the middle stage to detect potential molecular or behavioural changes at 12‐week‐old age (*n* = 9).

*C57BL/6J WT 12‐week‐old*: used for comparison with mutant mice at 12‐week‐old age (*n* = 9).

*Gba D409V KI 16‐week‐old*: mice monitored at the middle stage to detect potential molecular or behavioural changes at 16‐week‐old age (*n* = 9).

*C57BL/6J WT 16‐week‐old*: used for comparison with mutant mice at 16‐week‐old age (*n* = 9).

*Gba D409V KI 20‐week‐old*: mice monitored at late stage to detect potential molecular or behavioural changes at 20‐week‐old age (*n* = 9).

*C57BL/6J WT 20‐week‐old*: final time point reference group at 20‐week‐old age (*n* = 9).

At the end of each time point, behavioural assessments were done, and after the animals were sacrificed. Specifically, mice were deeply anaesthetised (sevoflurane overdose) and transcardially perfused with ice‐cold phosphate‐buffered saline (PBS) to remove circulating blood prior to brain collection. Brains or serum were then processed for molecular, biochemical and histological analyses (*n* = 9 for each technique: Behaviour/Histology; ELISA kits and RT‐qPCR respectively).

This design allowed the identification of time‐dependent alterations, with early and late phases distinguished by the emergence of redox imbalance and neurodegenerative hallmarks.

### 2.4 Behavioural Testing

Behavioural assessments were performed at each experimental endpoint. Prior to testing, mice were habituated to the testing environment for 5 min per day over two consecutive days. All behavioural evaluations were conducted by an experimenter blinded to the experimental group allocation.

#### 2.4.1 Elevated Plus Maze (EPM) Test

Anxiety‐like behaviour was assessed using the EPM test as previously described [26]. The apparatus consisted of two open arms, two closed arms and a central platform. Each mouse was placed on the central platform, facing an open arm, and observed for 5 min. Recorded parameters included duration of entries into open and closed arms, and time in the centre platform. Results were reported with time spent in the centre and closed arms expressed as seconds of the total session.

#### 2.4.2 Forced Swim Test (FST)

The FST was performed following the procedure previously indicated [27]. Mice were individually placed in a transparent glass cylinder (25 cm height, 14 cm diameter) filled with water (27°C, 20 cm depth) for a 6‐min session. The time spent immobile, considered as floating behaviour with minimal movements to keep the head above water, was recorded. Immobility was quantified during the final 4 min of the test.

### 2.5 Stereological Analysis

Unbiased stereological counting of TH^+^ dopaminergic neurons in the substantia nigra pars compacta (SNpc) was performed as previously described [28]. Sagittal sections (7 μm thick) were incubated overnight with a polyclonal anti‐tyrosine hydroxylase (TH) antibody (1:100; sc‐25269, Santa Cruz Biotechnology, Santa Cruz, CA, USA), followed by DAB‐based chromogenic detection. Sections were counterstained with cresyl violet for Nissl staining. Neurons were quantified using dedicated image analysis software under a Nikon Eclipse Ci‐L microscope, and representative images were acquired at 20× magnification. The stereological analysis was conducted by an experimenter blinded to the experimental group allocation.

### 2.6 Immunohistochemical Localization of Dopamine Transporter (DAT), α‐Synuclein, PARP1 and nNOS

Immunohistochemistry (IHC) was performed according to the procedure reported in our previous study [29], and specifically in SNpc. After paraffin removal through a graded series of alcohols, brain sections were incubated overnight with primary antibodies against DAT (1:100; sc‐14002, Santa Cruz Biotechnology), α‐synuclein (1:100; sc‐7011, Santa Cruz Biotechnology), PARP1 (1:100; sc‐74470, Santa Cruz Biotechnology) and nNOS (1:100; sc‐5302, Santa Cruz Biotechnology). Staining was carried out using the VECTASTAIN Universal Quick Kit, Peroxidase, R.T.U. (PK‐7800; Vector Laboratories, Burlingame, CA, USA). Sections were rinsed with PBS and subsequently exposed to the appropriate secondary antibody. The immunoreactive signal was visualized with 3,3′‐diaminobenzidine (DAB) as chromogen, and counterstained with Nuclear Fast Red. Quantification of immunoreactivity was performed using a computerized image analysis system by calculating the percentage of positive pixels relative to the total tissue area. Measurements were obtained from five random fields at 40× magnification. Microscopic analysis was carried out with a Nikon Eclipse Ci‐L microscope, and representative images are presented at 40× magnification. The immunohistochemical evaluations were conducted by an experimenter blinded to the experimental group allocation.

### 2.7 ELISA Kit

Nrf2, NOX2, HO‐1, MnSOD and NQO1 levels were determined as previously reported [30]. Tissue samples were homogenized and centrifuged at 14,000 *g* for 10 min at 4°C. Supernatants were then analysed using commercial ELISA kits following the manufacturer's instructions.

### 2.8 Quantitative Reverse Transcription PCR (RT‐qPCR)

Total RNA was isolated from midbrain tissue using the Trizol Reagent Kit (Life Technologies, Monza, Italy) for quantitative RT‐qPCR analysis, as previously described [31]. First‐strand cDNA was synthesized from 2.0 μg of RNA with the High‐Capacity cDNA Archive Kit (Applied Biosystems, Carlsbad, CA, USA). β‐actin mRNA was employed as an internal reference gene for normalization.

RT‐qPCR was carried out to assess the expression of Nrf2, NOX2, HO‐1, MnSOD and NQO1 using PowerUp SYBR Green Master Mix (Applied Biosystems, Carlsbad, CA, USA) on a QuantStudio 6 Flex Real‐Time PCR System (Applied Biosystems, Carlsbad, CA, USA). Amplification was quantified by determining cycle threshold (*C*_T_) values of each target gene relative to β‐actin. Fold changes in gene expression were calculated according to the $2^{-∆∆C_{t}}$ method, using the mean value of the control samples as calibrator. Primer sequences are reported in Table S1.

### 2.9 Malondialdehyde MDA

MDA levels in brain samples, used as an index of lipid peroxidation, were measured following previously described protocols [32].

### 2.10 Nitrate/Nitrite

Nitrate and nitrite concentrations in brain samples were quantified using the Griess reaction assay kit, and results were expressed as μmol of nitrite per mg of protein [32].

### 2.11 Statistical Analysis

All results are expressed as mean ± SD. Statistical analyses were carried out using GraphPad Prism 9 (GraphPad Software, San Diego, CA, USA). Data were analysed by two‐way ANOVA followed by Bonferroni's post hoc test. A *p*‐value < 0.05 was considered statistically significant [33].

## 3 Results

### 3.1 Parkinsonian‐Like Emotional Alterations in *Gba* D409V KI Mice

**FIGURE 2:**
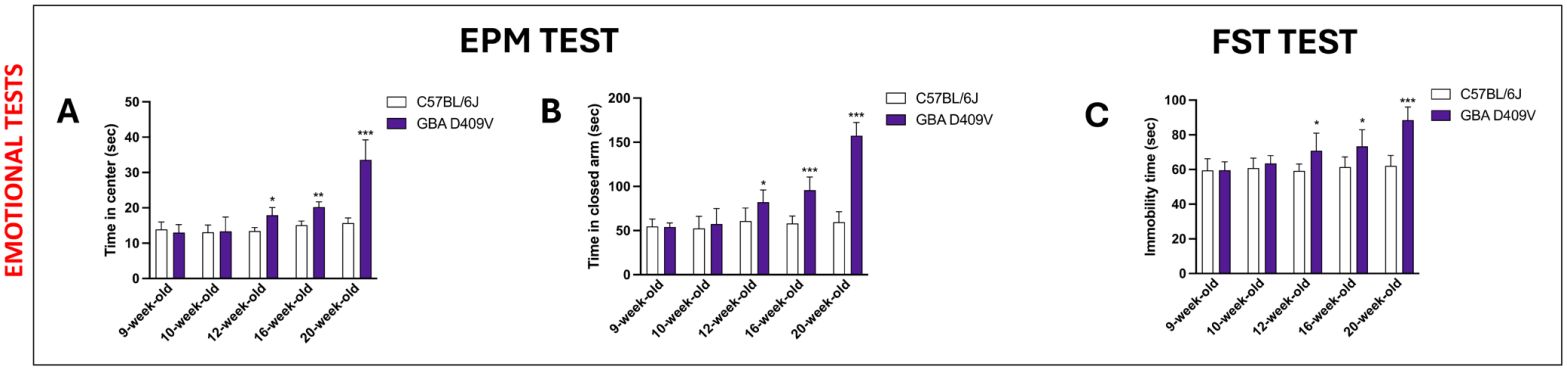
Evaluation of *Gba* D409V mutation on emotional behaviours. Emotional tests like EPM Test (A, B) and FST (C) were done. In each experimental group, the number of mice was *n* = 9. Values are means ± SD. Two‐way ANOVA test. *p < 0.05, **p < 0.01, ***p < 0.001 vs. C57BL/6J.

Emotional behaviour was assessed using the EPM and the FST tests. In the EPM, KI mice displayed increased time spent in the centre and in the closed arms, suggesting increased anxiety‐like behaviour (Figure 2A,B). Moreover, in the FST, *Gba* D409V animals exhibited increased immobility time compared to controls, consistent with depressive‐like behaviour (Figure 2C). Notably, these non‐motor alterations became evident at later stages of observation, thus resembling symptoms commonly reported in PD patients.

### 3.2 Time‐Course Evaluation of TH Expression in *Gba* D409V KI Mice

**FIGURE 3:**
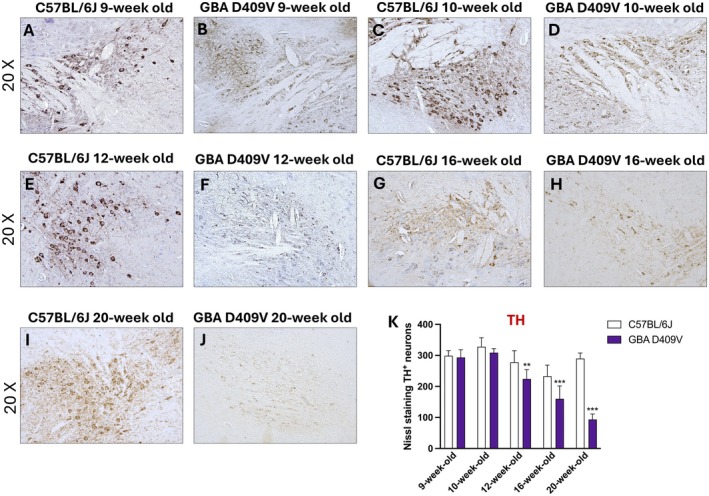
TH immunohistochemistry in the SNpc of *Gba* D409V KI mice. Representative immunohistochemical images show TH‐positive neurons in the SNpc of C57BL/6J and *Gba* D409V KI mice at different time points: 9‐week‐old (A, B), 10‐week‐old (C, D), 12‐week‐old (E, F), 16‐week‐old (G, H) and 20‐week‐old (I, J); score (K). Images are shown at 20× magnification. Each experimental group included *n* = 9 mice. Data are expressed as mean ± SD. Statistical analysis was performed by two‐way ANOVA. **p < 0.01, ***p < 0.001 vs. C57BL/6J.

To assess dopaminergic integrity, TH expression was evaluated at different time points by immunohistochemistry in SNpc. At early stages (9‐week‐old and 10‐week‐old), TH immunoreactivity in the SNpc of KI mice was comparable to that of WT controls (Figure 3A–D, score 3K), indicating preserved dopaminergic neurons in the initial phase of observation. The decrease in TH expression progressed over time, with changes becoming clearly detectable at 12‐week‐old (Figure 3E,F, score 3 K) and further exacerbated at 16‐week‐old and 20‐week‐old (Figure 3G–J, score 3K), where KI mice showed a marked and widespread reduction in TH‐positive neurons compared to age‐matched WT controls.

### 3.3 Time‐Course Assessment of DAT Expression in *Gba* D409V KI Mice

To further investigate the integrity of dopaminergic terminals, DAT expression was evaluated at different time points by immunohistochemistry in the SNpc.

**FIGURE 4:**
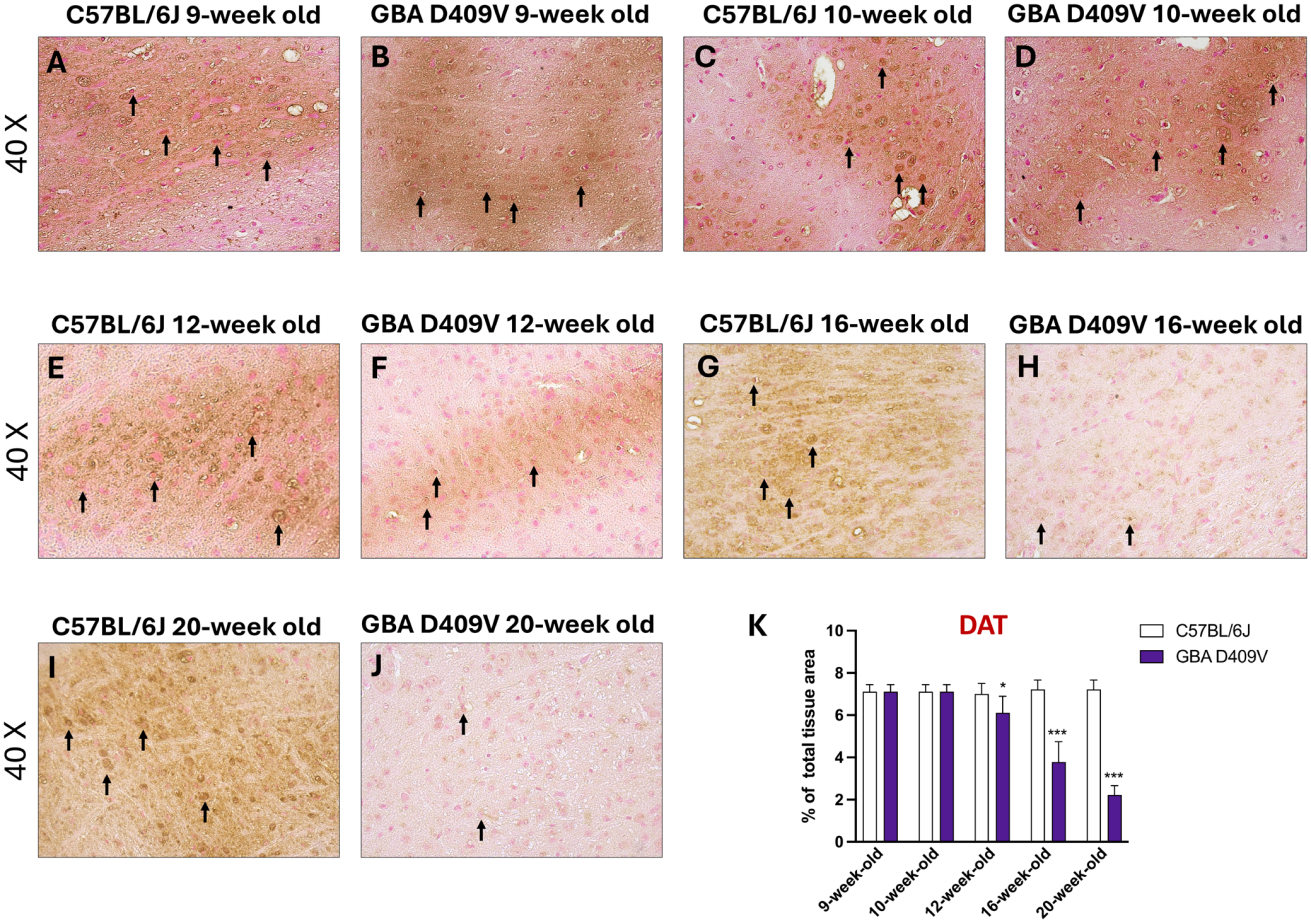
Representative immunohistochemical images show DAT expression in the SNpc of C57BL/6J and *Gba* D409V KI mice at different time points: 9‐week‐old (A, B), 10‐week‐old (C, D), 12‐week‐old (E, F), 16‐week‐old (G, H) and 20‐week‐old (I, J); score (K). Images are shown at 40× magnification. Each experimental group included *n* = 9 mice. Data are expressed as mean ± SD. Statistical analysis was performed by two‐way ANOVA. *p < 0.05, ***p < 0.001 vs. C57BL/6J.

In the SNpc of KI mice, DAT immunoreactivity was comparable to that of WT controls both at 9‐week‐old (Figure 4A,B, score 4K) and 10‐week‐old (Figure 4C,D, score 4K), indicating preserved dopaminergic terminals in the initial phase of observation. From 12‐week‐old onward, nevertheless, a reduction in DAT immunopositivity became evident in KI animals compared with controls (Figure 4E,F, score 4K). This reduction progressed over time, with a marked decrease observed at 16‐week‐old and 20‐week‐old (Figure 4G–J, score 4K).

### 3.4 Assessment of α‐Synuclein and p‐α‐Syn in *Gba* D409V KI Mice

**FIGURE 5:**
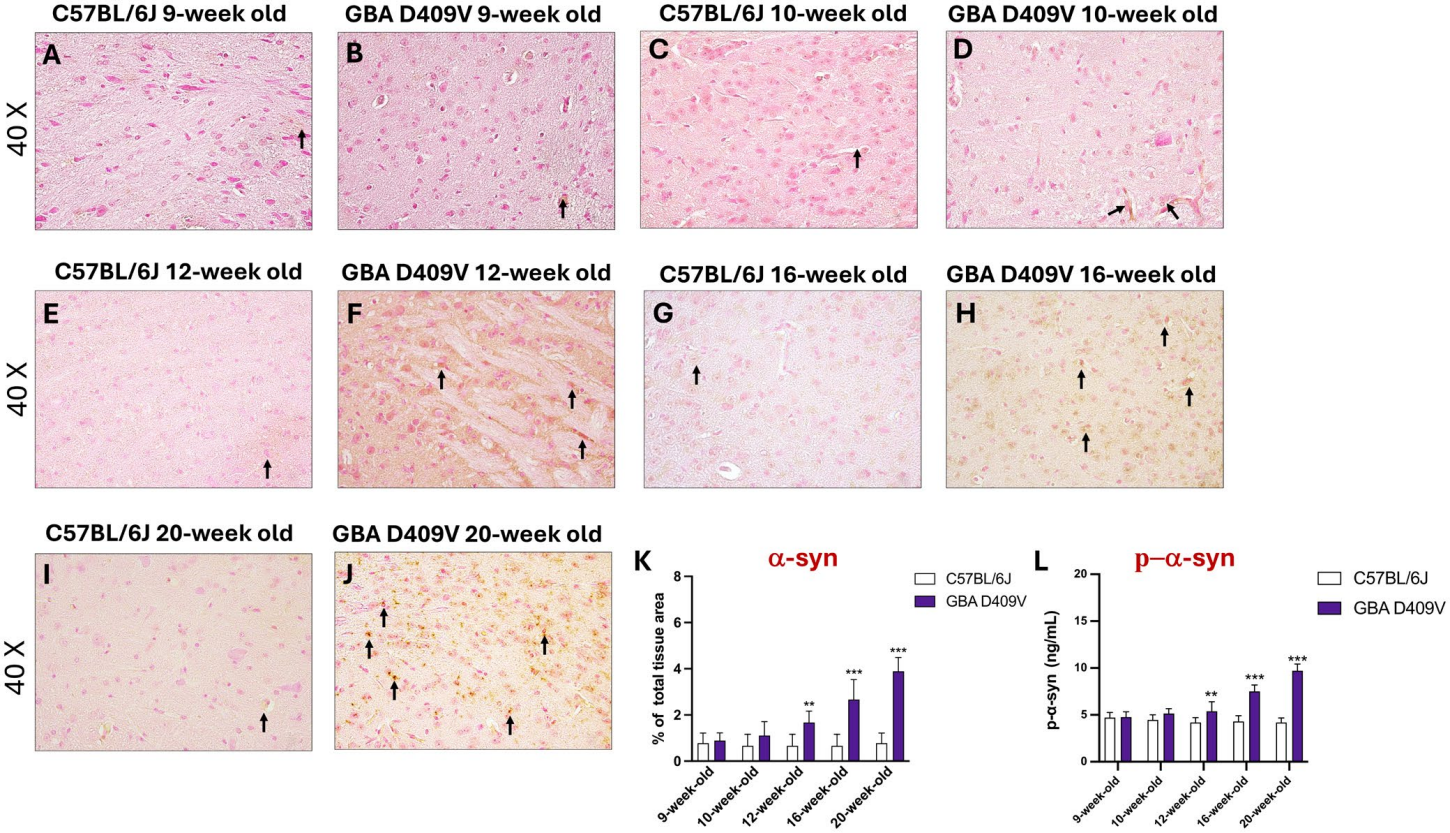
α‐syn and p‐α‐syn accumulation in the SNpc of *Gba* D409V KI mice. Representative immunohistochemical images show α‐synuclein immunoreactivity in the SNpc of C57BL/6J and *Gba* D409V KI mice at 9‐week‐old (A, B), 10‐week‐old (C, D), 12‐week‐old (E, F), 16‐week‐old (G, H) and 20‐week‐old (I, J); score (K). Levels of phosphorylated α‐syn were measured in tissue homogenates by ELISA at the indicated time points (L). Images are shown at 40× magnification. Each experimental group included *n* = 9 mice. Data are expressed as mean ± SD. Statistical analysis was performed by two‐way ANOVA. **p < 0.01, ***p < 0.001 vs. C57BL/6J.

The effect of the *Gba* D409V mutation on α‐syn pathology was examined in the SNpc. During the earliest observation windows (9‐week‐old and 10‐week‐old), α‐syn immunoreactivity was comparable between KI and WT mice, suggesting no overt accumulation at baseline (Figure 5A–D, score 5K).

From 12‐week‐old, KI animals began to exhibit an enhanced α‐syn positive signal compared to the respective control group (Figure 5E,F, score 5K). In subsequent analysis, α‐syn accumulations in SNpc became increasingly evident at 16‐week‐old and 20‐week‐old compared to WT (Figure 5G–J, score 5K).

In parallel, ELISA quantification of p‐α‐syn in SNpc homogenates confirmed the histological evidence. Indeed, levels of p‐α‐syn were comparable between groups at 9‐week‐old and 10‐week‐old but rose significantly in KI mice from 12‐week‐old onward (Figure 5L). The increase progressed in a time‐dependent manner, reaching maximal values at 16‐week‐old and especially 20‐week‐old (Figure 5L).

### 3.5 Evaluation of Oxidative Stress Markers in the Substantia Nigra of *Gba* D409V KI Mice

The temporal dynamics of oxidative stress regulation, in the context of GD‐correlating PD, were investigated by analysing the expression of Nrf2, NOX2, HO‐1, MnSOD and NQO1 in the SNpc through RT‐qPCR and ELISA.

**FIGURE 6:**
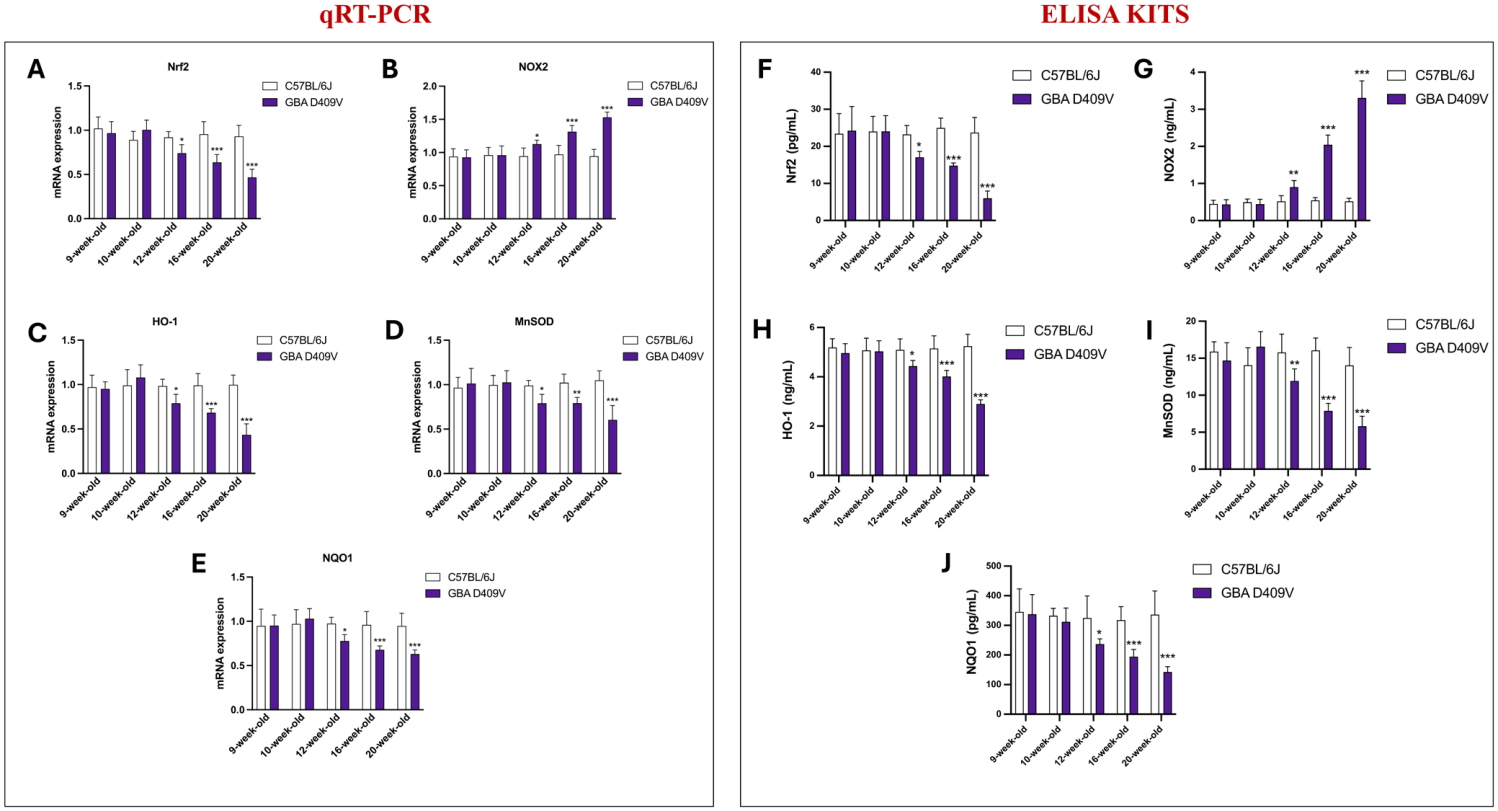
Temporal regulation of oxidative stress markers in the SNpc of *Gba* D409V KI and WT mice. Expression levels of Nrf2 (A, F), NOX2 (B, G), HO‐1 (C, H), MnSOD (D, I) and NQO1 (E, J) were quantified by RT‐qPCR and ELISA at different time points. Each experimental group included *n* = 9 mice. Data are expressed as mean ± SD. Statistical analysis was performed by two‐way ANOVA. *p < 0.05, **p < 0.01, ***p < 0.001 vs. C57BL/6J.

At 9‐week‐old and 10‐week‐old, no significant differences were detected between *Gba* D409V KI and WT mice, indicating that redox homeostasis was preserved during the initial phase of observation (Figure 6A–E for qRT‐PCR; Figure 6F–J for ELISA kits). From 12‐week‐old onward, however, a marked divergence in the redox profile became evident. Indeed, in *Gba* D409V KI animals, a progressive reduction in Nrf2 transcript and protein levels was observed (Figure 6A,F), accompanied by a concomitant downregulation of the downstream antioxidant targets HO‐1 (Figure 6C,H) and NQO1 (Figure 6E,J), as well as of MnSOD (Figure 6D,I). In contrast, NOX2 expression displayed a sustained upregulation, with significantly higher values compared to controls that became more pronounced at 16‐week‐old and 20‐week‐old (Figure 6B,G).

The concomitant decrease in Nrf2‐dependent antioxidant defences and the progressive induction of NOX2 highlight a shift towards a pro‐oxidant environment that emerges in later observations and intensifies during disease progression (Figure 6A–E for qRT‐PCR; Figure 6F–J for ELISA kits).

### 3.6 Temporal Upregulation of PARP1 and nNOS in the Substantia Nigra: Evidence of Redox Imbalance in the *Gba* D409V Model

The expression patterns of PARP1 and nNOS were evaluated in the SNpc of *Gba* D409V KI and WT mice by immunohistochemistry.

**FIGURE 7:**
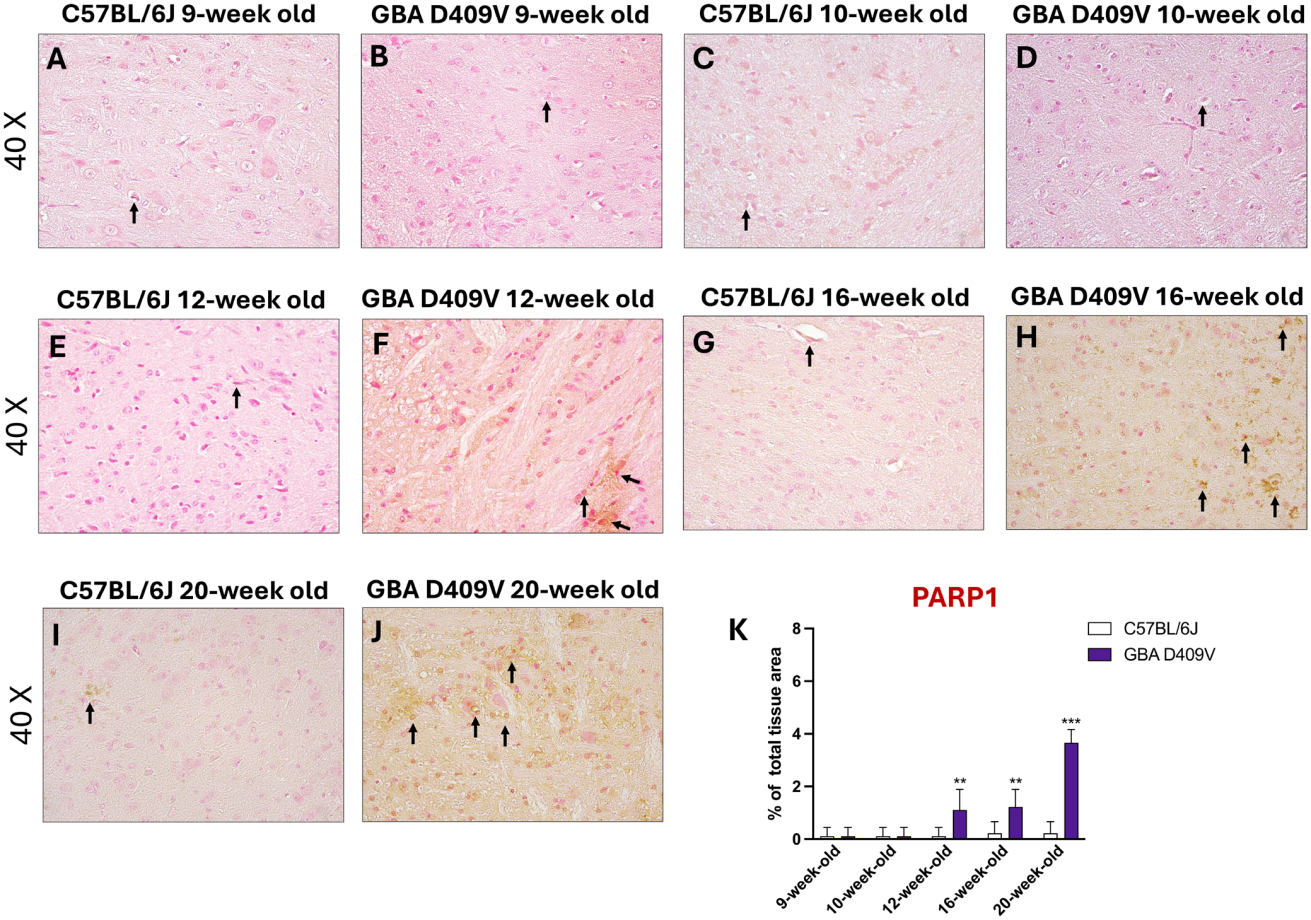
PARP1 immunoreactivity in the SNpc of *Gba* D409V KI mice. Representative immunohistochemical images show PARP1 expression in the SNpc of C57BL/6J and *Gba* D409V KI mice at 9‐week‐old (A, B), 10‐week‐old (C, D), 12‐week‐old (E, F), 16‐week‐old (G, H) and 20‐week‐old (I, J); score (K). Images are shown at 40× magnification. Each experimental group included *n* = 9 mice. Data are expressed as mean ± SD. Statistical analysis was performed by two‐way ANOVA. **p < 0.01, ***p < 0.001 vs. C57BL/6J.

At 9‐week‐old and 10‐week‐old, no appreciable differences in PARP1 staining were observed between *Gba* D409V KI and WT animals (Figure 7A–D, score 7K) indicating that DNA damage responses were not yet activated.

At 12‐week‐old and 16‐week‐old KI mice displayed a modest increase in PARP1 immunoreactivity (Figure 7F,H, score 7K) compared to control mice (Figure 7E,G, score 7K). By 20‐week‐old, PARP1 staining reached its maximum characterized by widespread and intense immunopositive signals in *Gb*a D409V mice (Figure 7J, score 7K) compared to C57BL/6J (Figure 7I, score 7K), thus pointing to sustained DNA damage and potential deleterious overactivation of repair pathways, which may contribute to neuronal dysfunction.

**FIGURE 8:**
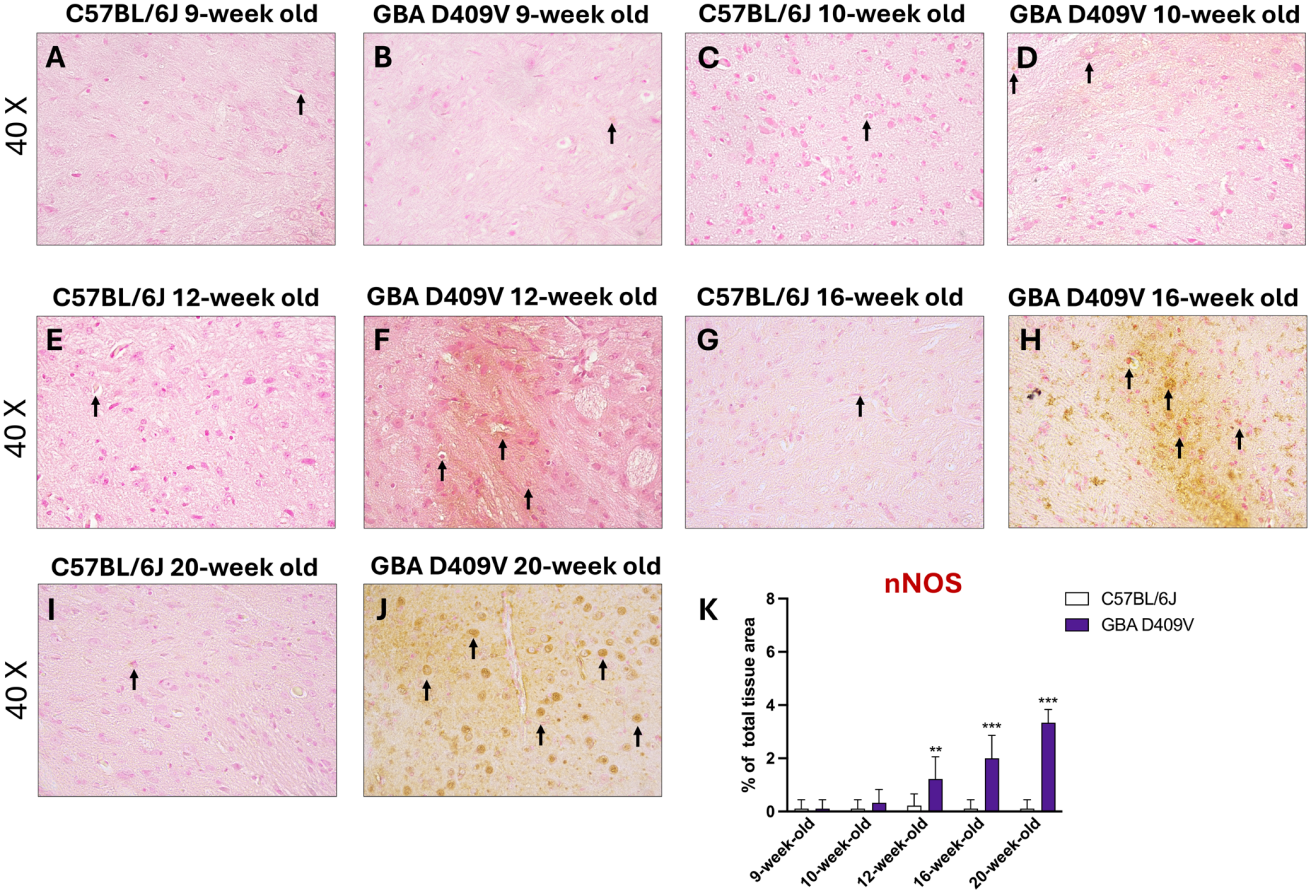
nNOS immunoreactivity in the SNpc of *Gba* D409V KI mice. Immunohistochemical staining illustrates nNOS expression in the SNpc of C57BL/6J and *Gba* D409V KI mice at 9‐week‐old (A, B), 10‐week‐old (C, D), 12‐week‐old (E, F), 16‐week‐old (G, H) and 20‐week‐old (I, J). Quantification of immunoreactivity is reported in panel (K). Images are shown at 40× magnification. Each group consisted of *n* = 9 animals. Values are presented as mean ± SD. Statistical comparisons were performed using two‐way ANOVA. **p < 0.01, ***p < 0.001 compared to C57BL/6J.

Likewise, at 9‐week‐old and 10‐week‐old, nNOS immunoreactivity remained comparable between groups, indicating preserved nitrosative balance in the early phase (Figure 8A–D, score 8K). Nevertheless, starting from 12‐week‐old a significant induction of nNOS immunopositivity was detected in *Gba* D409V mice compared to C57BL/6J strain (Figure 8E,F, score 8K).

From 16‐week‐old, however, KI mice exhibited a clear increase in nNOS immunopositive staining in the substantia nigra (Figure 8G,H, score 8K), reflecting the onset of nitrosative stress. This induction was further amplified at 20‐week‐old (Figure 8I–K), with intense nNOS immunolabelling, consistent with persistent nitric oxide overproduction.

### 3.7 Progressive Increase of Oxidative and Nitrosative Stress Markers in *Gba* D409V KI Mice

The levels of MDA and nitrate/nitrite were quantified in the SNpc of *Gba* D409V KI and WT mice using specific assay kits.

**FIGURE 9:**
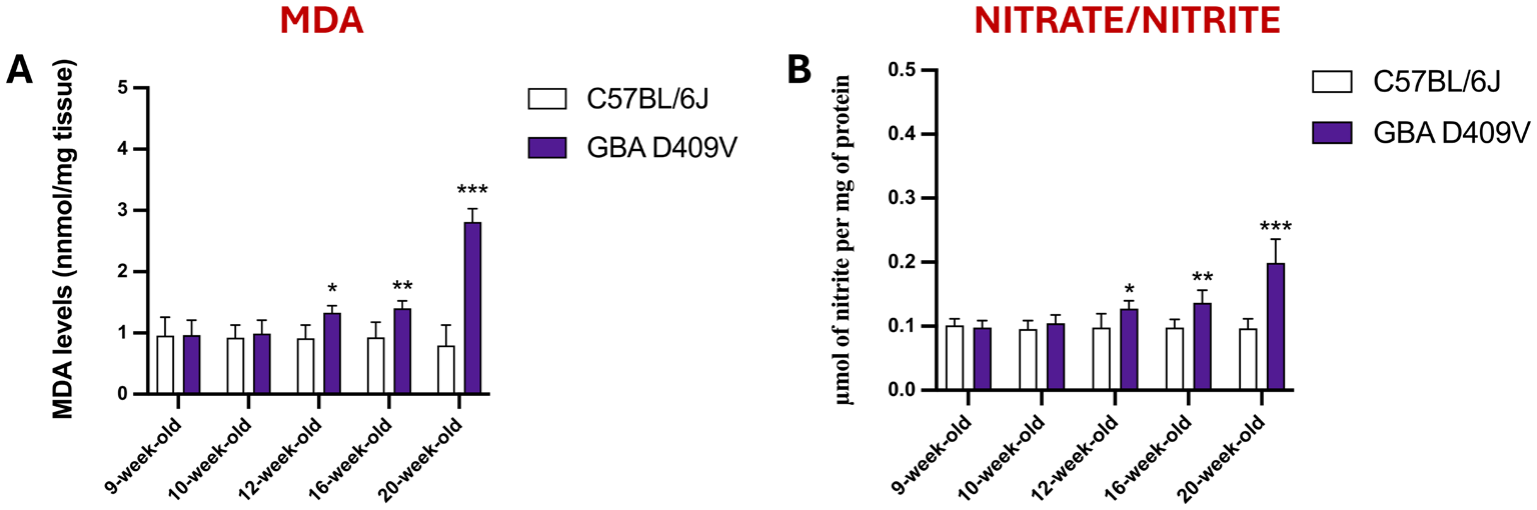
MDA and nitrate/nitrite levels in the SNpc of *Gba* D409V KI mice. Quantification of MDA (A) and nitrate/nitrite (B) concentrations was performed in the SNpc of C57BL/6J and *Gba* D409V KI mice at different time points using specific assay kits. Each experimental group included *n* = 9 mice. Data are expressed as mean ± SD. Statistical analysis was carried out by two‐way ANOVA. *p < 0.05, **p < 0.01, ***p < 0.001 vs. C57BL/6J.

At 9‐week‐old and 10‐week‐old, no significant differences were detected between groups, indicating that early oxidative and nitrosative stress responses were not yet evident (Figure 9A,B).

From 12‐week‐old onwards, *Gba* D409V mice exhibited a moderate but significant increase in MDA levels compared to WT, suggestive of enhanced lipid peroxidation (Figure 9A). This effect was further exacerbated at 16‐week‐old and culminated at 20‐week‐old, when MDA reached its maximum, pointing to sustained oxidative damage (Figure 9A).

Similarly, from 12‐week‐old nitrate/nitrite concentrations progressively rose in *Gba* D409V mice relative to controls, with the most pronounced elevations detected 16‐week‐old and 20‐week‐old (Figure 9B).

## 4 Discussion

Several studies across heterogeneous neurological and systemic disorders indicate that dysregulation of the Nrf2‐NOX2 axis constitutes a highly conserved determinant of redox imbalance [34, 35, 36]. In particular, in nitroglycerin‐induced migraine the selective NOX2 upregulation sustains neuronal and peripheral oxidative stress, as consequence the pharmacological Nrf2 activation mitigates symptom expression while attenuating microglial reactivity and inflammasome signalling [25]. Likewise, in neurodevelopmental conditions such as Autism Spectrum Disorder (ASD), impaired Nrf2 function has been associated with diminished antioxidant capacity, accumulation of oxidative damage products (lipid peroxidation, protein oxidation and DNA damage) and a shift towards chronic inflammatory tone [37].

Comparable patterns have also been documented in Duchenne muscular dystrophy, where altered Nrf2 activity correlates with disease severity and inflammatory cytokine burden, further linking Nrf2 tone to the modulation of peripheral tissue integrity [38]. All these evidences consistently emphasize that Nrf2 regulates a transcriptomic programme far beyond canonical antioxidant gene expression, including metabolic adaptation, proteostasis, mitochondrial biogenesis, autophagy and detoxification pathways. In this context, NOX2 is positioned upstream as a central source of ROS generation which, when unrestrained by Nrf2, drives feed‐forward oxidative stress, reinforcing pro‐inflammatory activation and propagates damage across neural and peripheral compartments.

Taken together, these observations support a generalisable model in which Nrf2 decline and NOX2 overactivation are not disease‐specific phenomena, but convergent elements of a shared pathological architecture that re‐appear across disorders characterized by oxidative stress, irrespective of the initiating insult. Crucially, pharmacological activation of Nrf2 in these settings is consistently associated with attenuation of oxidative injury, reduction of inflammatory signalling, preservation of neuronal integrity and, in extra‐neuronal tissues, maintenance of barrier and structural homeostasis.

This cross‐model reproducibility strengthens the mechanistic plausibility that the Nrf2–NOX2 axis is a biologically reliable target in conditions in which oxidative stress is pathophysiologically central.

On this basis, the present study specifically addressed whether the Nrf2–NOX2 axis is quantitatively altered in the context of *Gba*‐related parkinsonism in mice. *GBA* mutations are known to impair lysosomal function and to favour a cellular milieu characterized by lipid dyshomeostasis, proteostasis stress and increased susceptibility to oxidative injury [39]. Within this framework, Nrf2 represents the major endogenous transcriptional safeguard against redox imbalance, whereas NOX2 constitutes a principal enzymatic source of ROS generation [40]. However, despite extensive literature linking the Nrf2‐NOX2 axis to redox‐driven neurodegeneration in other conditions, this regulatory interface has not been systematically interrogated in GD‐associated PD. Accordingly, our study was designed to determine whether *Gba*‐related parkinsonism exhibits a definable disequilibrium between Nrf2 activity and NOX2‐dependent oxidant burden. Establishing the presence, or absence, of a measurable shift in this axis in the GD‐PD setting is a necessary step to clarify whether the Nrf2/NOX2 circuitry should be considered a biologically implicated pathway in this high‐risk genetic form of PD.

Emotional dysfunction is a well‐characterized clinical component of *GBA*‐associated parkinsonism [41], and previous cohort studies in humans have consistently shown that *GBA* mutation carriers exhibit earlier and more severe non‐motor manifestations compared to idiopathic PD, including anxiety and depressive traits [42]. Preclinical studies have also reported that lysosomal insufficiency in *GBA* loss‐of‐function models can disrupt dopaminergic circuitry and stress‐response networks, providing a mechanistic basis for early behavioural alterations even before overt nigrostriatal degeneration becomes fully established [13, 43].

In agreement with this framework, our KI model progressively developed non‐motor affective alterations, with emotional changes emerging in temporal continuity. These findings support the notion that the *Gba* D409V genotype is sufficient to drive a PD‐like behavioural phenotype under baseline conditions, without requiring additional toxic triggers. Importantly, the co‐occurrence of emotional alterations indicates that the consequences of *Gba* dysfunction extend beyond possible isolated motor pathway impairment and involve broader neural networks relevant to affect regulation. This clustering of behavioural phenotypes strengthens the interpretation that *GBA*‐related neurobiology generates a global vulnerability state, which may sensitize the system to redox‐dependent mechanisms examined in subsequent analyses.

Loss of dopaminergic integrity is a defining neuropathological feature of PD, and *GBA* mutation carriers consistently display both earlier dopaminergic terminal vulnerability and accelerated α‐syn pathology relative to sporadic cases [44, 45]. Previous works have shown that reduced GCase activity compromises lysosomal clearance pathways and favours α‐syn misfolding and aggregation, and viceversa, thereby increasing the burden on proteostasis mechanisms in nigrostriatal neurons [46, 47]. In this context, TH depletion and DAT downregulation are considered reliable correlates of progressive dopaminergic neuron dysfunction and terminal loss [48].

Here, the presence of preserved dopaminergic markers at the early observation windows, followed by marked loss of TH and DAT and a concomitant rise in α‐syn and p‐α‐syn towards the late phase (20‐week‐old), supports the interpretation that *Gba* loss‐of‐function does not simply accelerate an underlying degenerative programme. These observations strengthen the view that GCase deficiency establishes a pathological background in which dopaminergic neurons become progressively unable to maintain redox, proteostatic and synaptic homeostasis, a setting in which redox‐regulatory mechanisms such as the Nrf2/NOX2 axis could have a critical modulatory role.

As stated, Nrf2 is considered the major endogenous transcriptional regulator of antioxidant homeostasis [49], while NOX2 represents a key enzymatic source of ROS generation [50]. Therefore, establishing whether this axis is perturbed in experimental *Gba*‐related parkinsonism is critical to determine whether redox dysfunction constitutes a central mechanistic component of the phenotype.

In our model, a clear divergence between antioxidant defence and oxidant generation emerged after the initial observation window, with progressive downregulation of Nrf2 and its downstream antioxidant effectors (HO‐1 and MnSOD), accompanied by a sustained increase in NOX2 expression. This pattern strongly supports the interpretation that the *Gba* D409V genotype is sufficient to shift the substantia nigra microenvironment towards a pro‐oxidant state over time, rather than merely reflecting secondary consequences of neuronal impairment.

PARP1 hyperactivation and nitric oxide–derived reactive species have been repeatedly linked to redox‐mediated neurotoxicity in PD and related synucleinopathies [51, 52]. PARP1 upregulation is not simply a secondary marker of DNA injury, but a maladaptive response capable of depleting cellular NAD^+^ pools, impairing mitochondrial energy production, and promoting cell death pathways in dopaminergic neurons [53]. As well, nNOS induction increases the generation of reactive nitrogen species, which can react with superoxide to form peroxynitrite, drive protein nitration, and exacerbate membrane lipid oxidation [54]. In this biological setting, lipid peroxidation and elevated nitrate/nitrite levels are widely accepted biochemical indicators of sustained oxidative and nitrosative stress in dopaminergic circuit [55].

In the present study, the temporal increase over ageing in PARP1 immunopositivity and nNOS signals, together with progressive elevation of MDA and nitrate/nitrite levels, indicates an established redox disequilibrium in *Gba* D409V mice.

Collectively, these observations reinforce the view that Nrf2–NOX2 disequilibrium does not simply create ‘oxidative susceptibility’ but results in a progressive shift towards damage‐generating redox signalling involving DNA integrity, membrane lipid structure, and nitric oxide–related redox chemistry. Furthermore, these data support the interpretation that in the *Gba* D409V background, oxidative and nitrosative stress become functionally relevant components of disease evolution, providing a plausible mechanistic bridge between upstream redox dysregulation and the histopathological alterations of dopaminergic dysfunction.

This is an important point to address also in the treatment of other pathologies.

For instance, diabetes mellitus and related metabolic dysfunctions have been increasingly recognized as important risk factors for PD, influencing both disease onset and progression [56, 57]. Epidemiological and experimental studies suggest that insulin resistance, impaired glucose metabolism, and chronic systemic inflammation may exacerbate neuronal vulnerability, particularly within the dopaminergic system [58, 59, 60]. Notably, many of the molecular pathways implicated in diabetes‐related neurodegeneration overlap with those observed in *Gba1*‐associated Parkinsonism.

Oxidative and nitrosative stress represent a key mechanistic intersection between metabolic disorders and PD. Hyperglycemia and insulin resistance have been shown to promote excessive production of reactive oxygen and nitrogen species, mitochondrial dysfunction, and impaired cellular stress responses, processes that closely mirror the redox imbalance observed in GC [61, 62]. In this context, dysregulation of the Nrf2 antioxidant pathway and overactivation of NOX2 may act as converging mechanisms through which both lysosomal dysfunction and metabolic stress amplify oxidative damage and dopaminergic neurodegeneration.

Ageing further potentiates these effects, as it is associated with progressive metabolic decline, reduced antioxidant capacity and increased susceptibility to neuroinflammation. Thus, age‐related metabolic alterations may synergize with *Gba1* mutations to accelerate Parkinsonian pathology, providing a plausible biological link between GC–related Parkinsonism, diabetes, and sporadic PD. Although the present study did not directly assess diabetic phenotypes, our findings support the concept that systemic metabolic stress may represent an important modifier of disease progression in genetically vulnerable populations. So, these considerations highlight also the relevance of metabolic dysfunction as a potential contributing factor in *Gba1*‐associated Parkinsonism and underscore the importance of targeting redox and metabolic pathways as part of future therapeutic strategies.

In this field, the GD‐Parkinsonism mouse model may also be relevant to sporadic PD associated with type 2 diabetes. These observations support the hypothesis that chronic metabolic stress, also cooperating with genetic factors, may contribute to dopaminergic degeneration.

Although comprehensive longitudinal clinical studies following large cohorts of patients with both diabetes and Parkinsonism over extended periods remain challenging, available epidemiological data provide an important framework for interpreting experimental findings [63]. The convergence of lysosomal dysfunction, oxidative stress, impaired antioxidant defences, and mitochondrial abnormalities observed in *Gba1* D409V mice parallels key pathogenic features described in diabetic patients who develop PD.

Taken together, these considerations suggest that GD‐associated Parkinsonism and sporadic PD with metabolic comorbidities may share common pathogenic mechanisms. Integrating experimental models with epidemiological evidence may therefore be essential for identifying early biomarkers and developing therapeutic strategies targeting metabolic and redox dysregulation in PD.

While the present dataset provides robust evidence of progressive redox dysregulation in a *Gba1* KI background, several limitations should be acknowledged. First, this study focused on a single genetic model, the *Gba1* D409V variant, and therefore the findings cannot be directly generalized to other *GBA1* mutations that differ in biochemical severity and clinical penetrance. Future studies examining multiple *GBA* variants will be important to determine whether early redox failure represents a shared or mutation‐specific pathogenic mechanism.

Second, although cognitive impairment represents an important clinical feature of *GBA*‐related PD, the behavioural battery employed in the present study was designed a priori to capture early non‐motor features most directly linked to nigrostriatal dysfunction within the defined experimental time window. More comprehensive cognitive testing, such as Y‐maze or novel object recognition paradigms, was not included in the present study design and will be important to address in future investigations to further define the cognitive profile associated with early *GBA*‐related Parkinsonism. While this limits direct evaluation of early cognitive deficits, it does not detract from the central mechanistic conclusions drawn from convergent molecular, biochemical and neuropathological data.

Third, although our longitudinal analyses reveal a clear temporal association between Nrf2 impairment, sustained NOX2 induction, and disease progression, the present study did not include direct interventional modulation of these pathways. Consequently, while the data support a mechanistic role for the Nrf2‐NOX2 axis in disease evolution, pharmacological or genetic manipulation will be required to formally establish causality and therapeutic efficacy.

Finally, translation of these findings to human pathology will require validation in patient‐derived material, including post‐mortem tissue and accessible biofluids. Integration of experimental models with human biomarker studies will be essential to assess the clinical relevance of redox‐related signatures and to advance their potential use in early diagnosis or disease‐modifying strategies.

## 5 Conclusions

In summary, our work demonstrates that *GBA* loss‐of‐function is sufficient to trigger a progressive redox disequilibrium in vivo, emerging already at the earliest stages of disease development. By longitudinally monitoring the onset and evolution of Parkinson's disease–like features in *Gba1* D409V KI mice, we show that Nrf2 impairment and sustained NOX2 induction represent early drivers of oxidative and nitrosative stress, rather than downstream consequences of advanced neurodegeneration. This temporal mapping, when translated to human timescales corresponding to years of prodromal progression, supports the concept that redox failure is a foundational event in *GBA*‐associated neurodegeneration.

These findings reinforce the notion that *GBA*‐related parkinsonism is not merely a phenotypic variant of idiopathic PD, but a distinct biological entity in which lysosomal dysfunction and redox instability are mechanistically intertwined. Importantly, the identification of redox imbalance at such an early stage highlights the predictive potential of the Nrf2–NOX2 axis as a candidate biomarker of disease vulnerability and progression.

At the same time, species‐specific differences between mice and humans must be carefully considered when interpreting these findings in a translational context, particularly with respect to disease kinetics, metabolic regulation and therapeutic responsiveness. While the *Gba1* D409V mouse model provides a powerful platform to dissect early pathogenic mechanisms, integration with clinical, epidemiological, and human‐based experimental systems will be essential to improve the predictive value of preclinical research.

Future studies should determine whether strengthening Nrf2 signalling, inhibiting NOX2 activity, or adopting combinatorial approaches can modify these early disease trajectories and potentially delay PD onset in individuals carrying *GBA* mutations. The integration of temporal biomarkers with targeted interventions may ultimately shift this framework from descriptive pathophysiology towards mechanistic prediction and prevention, laying the groundwork for precision medicine strategies aimed at postponing, or mitigating, the emergence of *GBA*‐driven synucleinopathies.

## Author Contributions

**Alessio Ardizzone:** writing – original draft, data curation. **Giovanna Casili:** methodology. **Marika Lanza:** supervision. **Fabiola De Luca:** methodology. **Emanuela Esposito:** conceptualization, supervision. **Maria Bulzomì:** methodology. **Irene Paterniti:** supervision. **Anna Paola Capra:** methodology. **Michela Campolo:** writing – review and editing, supervision, conceptualization.

## Funding

This work was supported by the National Plan for NRRP Complementary Investments (PNC, established with the decree‐law 6 May 2021, n. 59, converted by law n. 101 of 2021) in the call for the funding of research initiatives for technologies and innovative trajectories in the health and care sectors (Directorial Decree n. 931 of 6 June 2022)—project n. PNC0000003—AdvaNced Technologies for Human‐centrEd Medicine (project acronym: ANTHEM). This work only reflects the authors' views and opinions; neither the Ministry for University and Research nor the European Commission can be considered responsible for them.

## Conflicts of Interest

The authors declare no conflicts of interest.

## Supporting information


**Table S1:** List of primers used for RT‐qPCR.

## Data Availability

All data generated or analysed during this study are included in this article.
