# Supplementary material for: Nrf2/NOX2 Pathway Dysregulation and Oxidative Stress Biomarkers in Gaucher Disease–Associated Parkinsonism: Insights Into a Potential Therapeutic Target
Source: J Cell Mol Med. 2026 Jun 2;30(11):e71225. doi: 10.1111/jcmm.71225 (PMC13239853; doi:10.1111/jcmm.71225)
Supplement: Supplementary file 1 — Table S1: List of primers used for RT‐qPCR. [file JCMM-30-e71225-s001.docx]

Supplementary Table 1. List of primers used for RT-qPCR

| Gene | Forward | Reverse |
| --- | --- | --- |
| ***Nrf2*** | CTGAACTCCTGGACGGGACTA | CGGTGGGTCTCCGTAAATGG |
| ***NOX2*** | CCAACTGGGATAACGAGTTCAA | TCAGGGCCACACAGGAAAA |
| ***HO-1*** | TGCTAGCCTGGTGCAAGATA | GCCAACAGGAAGCTGAGAGT |
| ***MnSOD*** | GTGGAGAACCCAAAGGAGAG | AACCTT GGACTCCCACAGAC |
| ***NQO1*** | GTCCATTCCAGCTGACAACCA | TTGCCCTGAGGCTCCTAATC |
| ***GAPDH*** | ACCTGCCAAGTATGATGACATCA | GGTCCTCAGTGTAGCCCAAGAT |
